# 4-Methyl-3-[4-(3-pyrid­yl)pyrimidin-2-yl­oxy]aniline

**DOI:** 10.1107/S1600536809023770

**Published:** 2009-06-27

**Authors:** Shi-Gui Tang, Cheng-Long Yang, Jun-Hua Chen, Sheng Bi, Cheng Guo

**Affiliations:** a College of Biology and Pharmacy Engineering, Nanjing University of Technology, Xinmofan Road No. 5 Nanjing, Nanjing 210009, People’s Republic of China; b College of Kang ni, Nanjing College of Engineering, Hongjing Road No. 1 Jiangning District, Nanjing, Nanjing 211167, People’s Republic of China; c College of Science, Nanjing University of Technology, Xinmofan Road No. 5 Nanjing, Nanjing 210009, People’s Republic of China; d Guangdong Petroleum College of Technology, Chisha Road No. 23 Nanhai District, Fushan, Fushan 528222, People’s Republic of China

## Abstract

In the title compound, C_16_H_14_N_4_O, there are inter­molecular N—H⋯N hydrogen bonds which may be effective in stabilizing the crystal. The title compound is an important medicament and is used in the synthesis of anti­tumour drugs.

## Related literature

For bond-length data, see: Allen *et al.* (1987 ▶)
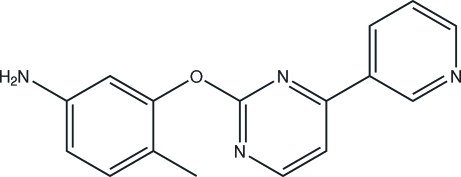


## Experimental

### 

#### Crystal data

C_16_H_14_N_4_O
*M* *_r_* = 278.31
Monoclinic, 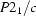

*a* = 8.5800 (17) Å
*b* = 20.360 (4) Å
*c* = 8.0780 (16) Å
β = 98.29 (3)°
*V* = 1396.4 (5) Å^3^
*Z* = 4
Mo *K*α radiation
μ = 0.09 mm^−1^
*T* = 293 K
0.20 × 0.20 × 0.10 mm

#### Data collection

Enraf–Nonius CAD-4 diffractometer
Absorption correction: ψ scan (North *et al.*, 1968 ▶) *T*_min_ = 0.983, *T*_max_ = 0.991
2698 measured reflections
2526 independent reflections
1587 reflections with *I* > 2σ(*I*)
*R*_int_ = 0.018
3 standard reflections every 200 reflections intensity decay: 1%

#### Refinement

*R*[*F* ^2^ > 2σ(*F* ^2^)] = 0.061
*wR*(*F* ^2^) = 0.170
*S* = 1.01
2526 reflections
190 parameters
H-atom parameters constrained
Δρ_max_ = 0.28 e Å^−3^
Δρ_min_ = −0.30 e Å^−3^

### 

Data collection: *CAD-4 EXPRESS* (Enraf–Nonius, 1989 ▶); cell refinement: *CAD-4 EXPRESS*; data reduction: *XCAD4* (Harms & Wocadlo, 1995 ▶); program(s) used to solve structure: *SHELXS97* (Sheldrick, 2008 ▶); program(s) used to refine structure: *SHELXL97* (Sheldrick, 2008 ▶); molecular graphics: *ORTEP-3 for Windows* (Farrugia, 1997 ▶); software used to prepare material for publication: *SHELXL97*.

## Supplementary Material

Crystal structure: contains datablocks global, I. DOI: 10.1107/S1600536809023770/pv2165sup1.cif

Structure factors: contains datablocks I. DOI: 10.1107/S1600536809023770/pv2165Isup2.hkl

Additional supplementary materials:  crystallographic information; 3D view; checkCIF report

## Figures and Tables

**Table 1:** Hydrogen-bond geometry (Å, °)

*D*—H⋯*A*	*D*—H	H⋯*A*	*D*⋯*A*	*D*—H⋯*A*
N1—H1*A*⋯N4^i^	0.86	2.47	3.214 (4)	145
N1—H1*B*⋯N2^ii^	0.86	2.43	3.166 (4)	144

Symmetry codes: (i) 
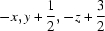
; (ii) 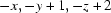.

**Fig. 1.**
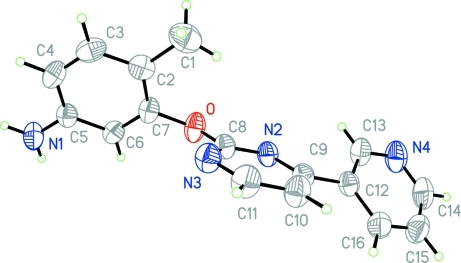
The molecular structure of (I), showing the atom-numbering scheme and displacement ellipsoids at the 50% probability level.

**Fig. 2.**
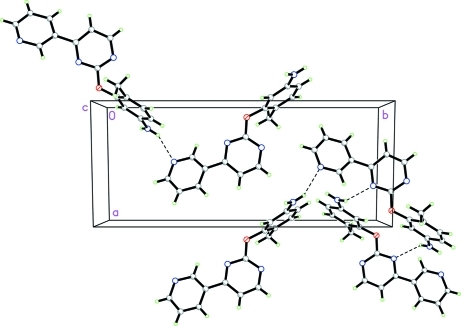
A packing diagram of (I). The intermolecular hydrogen bonds are shown as dashed lines.

## supplementary crystallographic information

### Comment

Some derivatives of benzenamine are important medical materials. We report here the crystal structure of the title compound, (I), which was synthesized by the reaction of *tert*-butyl-4-methyl-3-(4-(3-pyridinyl)pyrimidin-2 -yloxy)phenylcarbamate and dichloromethane with trifluoroacetic acid. The molecular structure of (I) is shown in Fig. 1. The bond lengths and angles in (I) are within normal ranges (Allen *et al.* 1987). The structure is stabilized by N—H···N type hydrogen bonds (Table 1 and Fig. 2).

### Experimental

In a three neck bottom flask containing dichloromethane (65 ml) and trifluoroacetic acid (20 ml) was added *tert*-butyl-4-methyl-3- (4-(3-pyridinyl)pyrimidin-2-yloxy)phenylcarbamate (7.5 g) at 273 K. After the addition of all chemicals, the flask was taken off the ice-water bath and the reaction was allowed to take place for 6 h at room temperature. Neutralized with sodium bicarbonate and separated the dichloromethane and aqueous layers. On evaporation of dichloromethane a solid product was obtained. Crystals of (I) suitable for X-ray diffraction were obstained by slow evaporation of a cyclohexane solution.

### Refinement

All H atoms bonded to the C atoms were placed geometrically at the distances of 0.93, 0.96 and 0.86 Å, for aryl, methyl and amino H-atoms, respectively, and were included in the refinement in riding motion approximation with *U*_iso_(H) = 1.2 or 1.5*U*_eq_ of the carrier atom.

### Crystal data

**Table:**

C_16_H_14_N_4_O	*F*(000) = 584
*M**_r_* = 278.31	*D*_x_ = 1.324 Mg m^−^^3^
Monoclinic, *P*2_1_/*c*	Mo *K*α radiation, λ = 0.71073 Å
Hall symbol: -P 2ybc	Cell parameters from 25 reflections
*a* = 8.5800 (17) Å	θ = 9–13°
*b* = 20.360 (4) Å	µ = 0.09 mm^−^^1^
*c* = 8.0780 (16) Å	*T* = 293 K
β = 98.29 (3)°	Block, colorless
*V* = 1396.4 (5) Å^3^	0.20 × 0.20 × 0.10 mm
*Z* = 4	

### Data collection

**Table:**

Enraf–Nonius CAD-4 diffractometer	1587 reflections with *I* > 2σ(*I*)
Radiation source: fine-focus sealed tube	*R*_int_ = 0.018
graphite	θ_max_ = 25.3°, θ_min_ = 2.0°
ω/2θ scans	*h* = −10→0
Absorption correction: ψ scan (North *et al.*, 1968)	*k* = 0→24
*T*_min_ = 0.983, *T*_max_ = 0.991	*l* = −9→9
2698 measured reflections	3 standard reflections every 200 reflections
2526 independent reflections	intensity decay: 1%

### Refinement

**Table:**

Refinement on *F*^2^	Secondary atom site location: difference Fourier map
Least-squares matrix: full	Hydrogen site location: inferred from neighbouring sites
*R*[*F*^2^ > 2σ(*F*^2^)] = 0.061	H-atom parameters constrained
*wR*(*F*^2^) = 0.170	*w* = 1/[σ^2^(*F*_o_^2^) + (0.06*P*)^2^ + 1.4*P*] where *P* = (*F*_o_^2^ + 2*F*_c_^2^)/3
*S* = 1.01	(Δ/σ)_max_ < 0.001
2526 reflections	Δρ_max_ = 0.28 e Å^−^^3^
190 parameters	Δρ_min_ = −0.30 e Å^−^^3^
0 restraints	Extinction correction: *SHELXL97* (Sheldrick, 2008)
Primary atom site location: structure-invariant direct methods	Extinction coefficient: 0.017 (4)

### Special details

**Table:**

Geometry. All e.s.d.'s (except the e.s.d. in the dihedral angle between two l.s. planes) are estimated using the full covariance matrix. The cell e.s.d.'s are taken into account individually in the estimation of e.s.d.'s in distances, angles and torsion angles; correlations between e.s.d.'s in cell parameters are only used when they are defined by crystal symmetry. An approximate (isotropic) treatment of cell e.s.d.'s is used for estimating e.s.d.'s involving l.s. planes.
Refinement. Refinement of *F*^2^ against ALL reflections. The weighted *R*-factor *wR* and goodness of fit *S* are based on *F*^2^, conventional *R*-factors *R* are based on *F*, with *F* set to zero for negative *F*^2^. The threshold expression of *F*^2^ > σ(*F*^2^) is used only for calculating *R*-factors(gt) *etc*. and is not relevant to the choice of reflections for refinement. *R*-factors based on *F*^2^ are statistically about twice as large as those based on *F*, and *R*- factors based on ALL data will be even larger.

### Fractional atomic coordinates and isotropic or equivalent isotropic displacement parameters (Å^2^)

**Table:**

	*x*	*y*	*z*	*U*_iso_*/*U*_eq_	
O	0.1194 (2)	0.51325 (11)	0.7760 (3)	0.0508 (6)	
N1	−0.2002 (3)	0.66654 (13)	1.0241 (4)	0.0543 (8)	
H1A	−0.2537	0.7024	1.0186	0.065*	
H1B	−0.1955	0.6419	1.1111	0.065*	
C1	0.1120 (5)	0.5926 (2)	0.4732 (5)	0.0680 (11)	
H1C	0.1658	0.5515	0.4963	0.102*	
H1D	0.1862	0.6258	0.4525	0.102*	
H1E	0.0335	0.5879	0.3765	0.102*	
N2	0.3246 (3)	0.44734 (12)	0.7623 (3)	0.0416 (7)	
C2	0.0341 (4)	0.61219 (17)	0.6213 (4)	0.0460 (8)	
N3	0.3632 (3)	0.56324 (13)	0.7919 (4)	0.0497 (7)	
C3	−0.0526 (4)	0.66983 (17)	0.6214 (4)	0.0498 (9)	
H3B	−0.0593	0.6973	0.5285	0.060*	
C4	−0.1290 (4)	0.68813 (16)	0.7523 (4)	0.0480 (9)	
H4A	−0.1857	0.7272	0.7468	0.058*	
N4	0.4792 (4)	0.26228 (15)	0.6373 (4)	0.0649 (9)	
C5	−0.1216 (3)	0.64847 (14)	0.8928 (4)	0.0405 (8)	
C6	−0.0318 (3)	0.59152 (14)	0.8984 (4)	0.0395 (8)	
H6A	−0.0230	0.5645	0.9921	0.047*	
C7	0.0441 (3)	0.57516 (15)	0.7653 (4)	0.0403 (8)	
C8	0.2772 (4)	0.50942 (15)	0.7753 (4)	0.0412 (8)	
C9	0.4799 (4)	0.43896 (15)	0.7646 (4)	0.0406 (8)	
C10	0.5823 (4)	0.49200 (17)	0.7776 (5)	0.0522 (9)	
H10A	0.6898	0.4863	0.7773	0.063*	
C11	0.5179 (4)	0.55349 (17)	0.7909 (5)	0.0556 (10)	
H11A	0.5845	0.5898	0.7995	0.067*	
C12	0.5341 (4)	0.37050 (15)	0.7517 (4)	0.0411 (8)	
C13	0.4408 (4)	0.32551 (17)	0.6553 (5)	0.0526 (9)	
H13A	0.3447	0.3400	0.5990	0.063*	
C14	0.6177 (5)	0.24293 (19)	0.7200 (5)	0.0630 (11)	
H14A	0.6476	0.1993	0.7104	0.076*	
C15	0.7182 (5)	0.28438 (19)	0.8184 (5)	0.0619 (10)	
H15A	0.8136	0.2687	0.8737	0.074*	
C16	0.6774 (4)	0.34860 (18)	0.8347 (5)	0.0531 (9)	
H16A	0.7445	0.3772	0.9004	0.064*	

### Atomic displacement parameters (Å^2^)

**Table:**

	*U*^11^	*U*^22^	*U*^33^	*U*^12^	*U*^13^	*U*^23^
O	0.0398 (13)	0.0379 (12)	0.0783 (18)	−0.0001 (10)	0.0208 (11)	−0.0024 (11)
N1	0.066 (2)	0.0395 (16)	0.0609 (19)	0.0056 (14)	0.0227 (16)	−0.0018 (14)
C1	0.069 (3)	0.083 (3)	0.055 (2)	−0.006 (2)	0.019 (2)	0.000 (2)
N2	0.0410 (16)	0.0358 (14)	0.0497 (17)	−0.0021 (12)	0.0123 (12)	−0.0049 (12)
C2	0.0391 (18)	0.053 (2)	0.046 (2)	−0.0059 (16)	0.0087 (15)	0.0002 (16)
N3	0.0463 (17)	0.0412 (16)	0.0619 (19)	−0.0045 (13)	0.0084 (14)	−0.0053 (14)
C3	0.0455 (19)	0.052 (2)	0.051 (2)	−0.0074 (17)	0.0033 (16)	0.0154 (17)
C4	0.0435 (19)	0.0358 (18)	0.065 (2)	0.0023 (15)	0.0084 (17)	0.0086 (16)
N4	0.062 (2)	0.0462 (18)	0.090 (3)	−0.0014 (16)	0.0242 (18)	−0.0145 (17)
C5	0.0369 (17)	0.0353 (17)	0.050 (2)	−0.0061 (14)	0.0070 (14)	−0.0037 (15)
C6	0.0381 (17)	0.0328 (17)	0.048 (2)	−0.0047 (14)	0.0077 (15)	0.0052 (14)
C7	0.0340 (17)	0.0313 (16)	0.057 (2)	−0.0029 (13)	0.0106 (15)	−0.0013 (15)
C8	0.0406 (18)	0.0393 (18)	0.0453 (19)	−0.0014 (15)	0.0116 (14)	−0.0034 (15)
C9	0.0393 (18)	0.0424 (18)	0.0414 (18)	−0.0007 (14)	0.0101 (14)	−0.0043 (14)
C10	0.0387 (18)	0.048 (2)	0.071 (3)	−0.0050 (16)	0.0125 (17)	−0.0057 (18)
C11	0.045 (2)	0.045 (2)	0.076 (3)	−0.0100 (16)	0.0074 (18)	−0.0075 (18)
C12	0.0387 (18)	0.0407 (18)	0.0462 (19)	−0.0019 (14)	0.0143 (15)	−0.0032 (15)
C13	0.0426 (19)	0.047 (2)	0.070 (2)	−0.0003 (16)	0.0138 (17)	−0.0118 (18)
C14	0.070 (3)	0.044 (2)	0.081 (3)	0.010 (2)	0.033 (2)	0.000 (2)
C15	0.058 (2)	0.057 (2)	0.072 (3)	0.018 (2)	0.012 (2)	0.006 (2)
C16	0.050 (2)	0.054 (2)	0.056 (2)	0.0034 (17)	0.0087 (17)	−0.0060 (17)

### Geometric parameters (Å, °)

**Table:**

O—C8	1.357 (4)	N4—C14	1.336 (5)
O—C7	1.413 (4)	N4—C13	1.342 (4)
N1—C5	1.387 (4)	C5—C6	1.389 (4)
N1—H1A	0.8600	C6—C7	1.376 (4)
N1—H1B	0.8600	C6—H6A	0.9300
C1—C2	1.506 (5)	C9—C10	1.387 (4)
C1—H1C	0.9600	C9—C12	1.478 (4)
C1—H1D	0.9600	C10—C11	1.379 (5)
C1—H1E	0.9600	C10—H10A	0.9300
N2—C8	1.337 (4)	C11—H11A	0.9300
N2—C9	1.340 (4)	C12—C13	1.381 (5)
C2—C7	1.379 (5)	C12—C16	1.386 (5)
C2—C3	1.389 (5)	C13—H13A	0.9300
N3—C8	1.317 (4)	C14—C15	1.375 (5)
N3—C11	1.344 (4)	C14—H14A	0.9300
C3—C4	1.374 (5)	C15—C16	1.365 (5)
C3—H3B	0.9300	C15—H15A	0.9300
C4—C5	1.387 (4)	C16—H16A	0.9300
C4—H4A	0.9300		
			
C8—O—C7	119.9 (2)	C6—C7—O	115.6 (3)
C5—N1—H1A	120.0	C2—C7—O	120.8 (3)
C5—N1—H1B	120.0	N3—C8—N2	128.5 (3)
H1A—N1—H1B	120.0	N3—C8—O	119.8 (3)
C2—C1—H1C	109.5	N2—C8—O	111.7 (3)
C2—C1—H1D	109.5	N2—C9—C10	121.3 (3)
H1C—C1—H1D	109.5	N2—C9—C12	116.2 (3)
C2—C1—H1E	109.5	C10—C9—C12	122.5 (3)
H1C—C1—H1E	109.5	C11—C10—C9	117.1 (3)
H1D—C1—H1E	109.5	C11—C10—H10A	121.4
C8—N2—C9	115.6 (3)	C9—C10—H10A	121.4
C7—C2—C3	115.5 (3)	N3—C11—C10	122.8 (3)
C7—C2—C1	123.0 (3)	N3—C11—H11A	118.6
C3—C2—C1	121.5 (3)	C10—C11—H11A	118.6
C8—N3—C11	114.6 (3)	C13—C12—C16	117.5 (3)
C4—C3—C2	123.0 (3)	C13—C12—C9	120.1 (3)
C4—C3—H3B	118.5	C16—C12—C9	122.4 (3)
C2—C3—H3B	118.5	N4—C13—C12	124.5 (3)
C3—C4—C5	120.1 (3)	N4—C13—H13A	117.8
C3—C4—H4A	120.0	C12—C13—H13A	117.8
C5—C4—H4A	120.0	N4—C14—C15	123.1 (3)
C14—N4—C13	116.3 (3)	N4—C14—H14A	118.4
C4—C5—N1	120.1 (3)	C15—C14—H14A	118.4
C4—C5—C6	118.2 (3)	C16—C15—C14	119.7 (4)
N1—C5—C6	121.7 (3)	C16—C15—H15A	120.2
C7—C6—C5	119.9 (3)	C14—C15—H15A	120.2
C7—C6—H6A	120.0	C15—C16—C12	118.9 (4)
C5—C6—H6A	120.0	C15—C16—H16A	120.5
C6—C7—C2	123.2 (3)	C12—C16—H16A	120.5
			
C7—C2—C3—C4	−2.5 (5)	C7—O—C8—N2	−171.2 (3)
C1—C2—C3—C4	178.0 (3)	C8—N2—C9—C10	−0.8 (5)
C2—C3—C4—C5	0.0 (5)	C8—N2—C9—C12	179.5 (3)
C3—C4—C5—N1	−179.2 (3)	N2—C9—C10—C11	1.1 (5)
C3—C4—C5—C6	1.9 (5)	C12—C9—C10—C11	−179.2 (3)
C4—C5—C6—C7	−1.3 (4)	C8—N3—C11—C10	−1.5 (5)
N1—C5—C6—C7	179.8 (3)	C9—C10—C11—N3	0.1 (6)
C5—C6—C7—C2	−1.2 (5)	N2—C9—C12—C13	34.5 (4)
C5—C6—C7—O	−174.0 (3)	C10—C9—C12—C13	−145.2 (3)
C3—C2—C7—C6	3.1 (5)	N2—C9—C12—C16	−145.1 (3)
C1—C2—C7—C6	−177.4 (3)	C10—C9—C12—C16	35.2 (5)
C3—C2—C7—O	175.4 (3)	C14—N4—C13—C12	0.1 (5)
C1—C2—C7—O	−5.0 (5)	C16—C12—C13—N4	0.2 (5)
C8—O—C7—C6	−119.7 (3)	C9—C12—C13—N4	−179.3 (3)
C8—O—C7—C2	67.4 (4)	C13—N4—C14—C15	−0.3 (6)
C11—N3—C8—N2	1.9 (5)	N4—C14—C15—C16	0.0 (6)
C11—N3—C8—O	179.5 (3)	C14—C15—C16—C12	0.4 (6)
C9—N2—C8—N3	−0.8 (5)	C13—C12—C16—C15	−0.5 (5)
C9—N2—C8—O	−178.5 (3)	C9—C12—C16—C15	179.1 (3)
C7—O—C8—N3	10.9 (5)		

### Hydrogen-bond geometry (Å, °)

**Table:**

*D*—H···*A*	*D*—H	H···*A*	*D*···*A*	*D*—H···*A*
N1—H1A···N4^i^	0.86	2.47	3.214 (4)	145
N1—H1B···N2^ii^	0.86	2.43	3.166 (4)	144

Symmetry codes: (i) −*x*, *y*+1/2, −*z*+3/2; (ii) −*x*, −*y*+1, −*z*+2.
